# Sensory Gating Scales and Premonitory Urges in Tourette Syndrome

**DOI:** 10.1100/tsw.2011.57

**Published:** 2011-03-22

**Authors:** Ashley N. Sutherland Owens, Euripedes C. Miguel, Neal R. Swerdlow

**Affiliations:** ^1^ Department of Psychiatry UCSD School of Medicine La Jolla CA USA; ^2^ Departamento de Psiquiatria da Faculdade de Medicina da USP Sao Paulo SP Brazil

**Keywords:** premonitory urge, sensory gating, tic, Tourette syndrome

## Abstract

Sensory and sensorimotor gating deficits characterize both Tourette syndrome (TS) and schizophrenia. Premonitory urges (PU) in TS can be assessed with the University of Sao Paulo Sensory Phenomena Scale (USP-SPS) and the Premonitory Urge for Tics Scale (PUTS). In 40 subjects (TS: n = 18; healthy comparison subjects [HCS]: n = 22), we examined the relationship between PU scores and measures of sensory gating using the USP-SPS, PUTS, Sensory Gating Inventory (SGI), and Structured Interview for Assessing Perceptual Anomalies (SIAPA), as well symptom severity scales. SGI, but not SIAPA, scores were elevated in TS subjects (*p* < 0.0003). In TS subjects, USP-SPS and PUTS scores correlated significantly with each other, but not with the SGI or SIAPA; neither PU nor sensory gating scales correlated significantly with symptom severity. TS subjects endorse difficulties in sensory gating and the SGI may be valuable for studying these clinical phenomena.

## INTRODUCTION

Tourette syndrome (TS) is one of several brain disorders characterized by symptoms that suggest failures in the automatic "gating" of sensory stimuli. In TS, intrusive sensory information is often experienced as pressure or discomfort, at or below the skin level, or as a mental sensation[1]. These "sensory tics" are often followed by, and may trigger, motor and vocal tics that historically have defined this disorder. A variation of the "sensory tic" in TS takes the form of uncomfortable urges or mental states[2,3]. This sensory or mental discomfort offers TS patients an opportunity to identify an imminent motor or vocal tic, and to intervene using tools such as behavioral therapy. Thus, a more complete understanding of sensory phenomena may have direct clinical applications in TS and valid methods of quantifying these experiences in TS have become increasingly important for treatment development[4]. Two such scales are the Premonitory Urge for Tics Scale (PUTS[5]) and the University of Sao Paulo (USP) Sensory Phenomena Scale (USP-SPS[6,7]). The PUTS is a brief self-report of the frequency of specific pre–tic related sensory symptoms, while the USP-SPS assesses the frequency and severity of sensory phenomena that precede, accompany, or follow tics and other repetitive behaviors, such as compulsions or rituals.

Impaired sensory gating also characterizes schizophrenia and has been quantified in scales, including the Structured Interview for Assessing Perceptual Anomalies (SIAPA and SIAPA-CV[8,9]) and the Sensory Gating Inventory (SGI[10]). The aim of this preliminary study was to determine whether these scales of sensory gating provide novel information regarding TS symptoms, which might inform us about the role of sensory gating deficits in the genesis of symptoms in TS.

## METHODS

Methods were approved by the UCSD and SDSU Institutional Review Boards; the study was conducted at the UCSD Medical Center. Twenty TS and 22 age-matched healthy comparison subjects (HCS) passed phone screens. Exclusion criteria for all subjects included serious medical, neurologic, or psychiatric illness (other than TS, OCD, or ADHD); schizophrenia in a first-degree relative; loss of consciousness (>1 min); current substance abuse or dependence; pregnancy; or known hearing loss. HCS were also excluded for a history of mental illness or psychotropic medication use. Presence or absence of sensory phenomena was not used as a basis for study inclusion/exclusion. All screening questions were then repeated in person and all adults provided urine for toxicology; two adult TS subjects were excluded for positive toxicology. Medications (n) in TS subjects included: antidepressants (8), alpha-norepinephrine agonists (5), benzodiazepines (3), anticonvulsants (3), dopamine agonists, (2) dopamine partial agonist/antagonists (2), and stimulants (2). Participants underwent structured and semi-structured clinical interviews for three purposes: (1) global clinical diagnosis: Structured Clinical Interview for DSM-IV Axis I Disorders (SCID-I/NP[11]), Diagnostic Interview Schedule for Children, computerized version IV (C-DISC-IV[12]); (2) general symptom severity for TS and OCD: Yale Global Tic Severity Scale, adult and child versions (YGTSS and CYGTSS[13]), Yale-Brown Obsessive Compulsive Scale, adult and child versions (YBOCS and CYBOCS[14,15,16]); and (3) specific symptom severity related to sensory phenomena and premonitory urges: USP-SPS[6,7], PUTS[5], SGI[10], and the SIAPA (adult and child versions)[8,9]. Nine TS subjects carried diagnoses of OCD and two carried ADHD diagnoses.

Scale scores were treated as continuous variables and group comparisons (TS vs. HCS) used mixed-design ANOVAs; items best accounting for group identity were detected via stepwise discriminant function analysis. Relationships between scale scores or between scores and age were assessed by simple regression analyses. Alpha was 0.05.

## RESULTS

YGTSS/CYGTSS scores suggested mild-to-moderate symptoms; YBOCS/ CYBOCS scores suggested mild symptoms (Table 1). Sensory phenomena were documented in the USP-SPS (M = 8.5; SD = 3.7) and PUTS total scores (M = 25.8; SD = 5.0), with a statistically significant positive correlation between USP-SPS and PUTS total scores ($R^2 = 0.349$; $p < 0.01$). Primary symptoms included uncomfortable physical sensations; “just-right” experiences triggered by visual, auditory, or tactile sensations; feelings of incompleteness/need to feel “just right”; and feelings of energy building up within the body that need to be released. USP-SPS scores tended to increase with age, but did not differ significantly across age groups (F = 1.91; df 1,14; ns; $R^2 = 0.192$; $p < 0.08$). ANOVA of the PUTS total score revealed a significant effect of age (F = 5.77; df 1,16; $p < 0.03$), with higher scores in adults vs. minors, confirmed by simple regression of PUTS total score and age ($R^2 = 0.434$; $p < 0.005$). Neither USP-SPS nor PUTS scores exhibited sex differences. There were no significant correlations between clinical PU scales (USP-SPS or PUTS) and scales of tics (motor [r’s = 0.20, -0.37], vocal [r’s = 0.0.20, 0.05], global [r’s = 0.17, -0.21]), obsessions (r’s = 0.00, -0.28), or compulsions (r’s = 0.21, -0.20).

**TABLE 1**

**A. Group Characteristics (M [SD])**

| | | TS | HCS |
|---|---|---|---|
| N (age, years) | >18 | 9 (25.5 [9.1]) | 10 (23.5 [5.9]) |
| | <18 | 9 (13.2 [2.9]) | 12 (13.2 [2.2]) |
| Male:Female | >18 | 6:3 | 8:2 |
| | <18 | 7:2 | 9:3 |
| YGTSS/CYGTSS Total | | 24.2 (8.0) | |
| Motor Subscale | | 13.7 (4.4) | |
| Vocal Subscale | | 10.5 (6.2) | |
| Global Subscale | | 40.4 (15.4) | |
| YBOCS/CYBOCS Total | | 10.6 (7.1) | |
| Obsession Subscale | | 5.7 (4.6) | |
| Compulsion Subscale | | 4.9 (3.2) | |

**B. TS Subject Summary Demographics and Scale Scores (Totals)**

| Age | Sex | Co-Dx | YGTSS/CYGTSS | YBOCS/CYBOCS | USP-SPS | PUTS | SGI | SIAPA |
|---|---|---|---|---|---|---|---|---|
| 10 | F | OCD | 31 | 27 | 3 | 17 | 125 | 17 |
| 10 | M | None | 5 | 0 | 8 | 29 | 60 | 17 |
| 11 | F | None | 21 | 6 | 10 | 25 | 141 | 41 |
| 11 | M | None | 36 | 16 | 9 | 18 | 45 | 15 |
| 12 | M | None | 28 | 15 | 11 | 27 | 73 | 21 |
| 16 | M | ADHD | 18 | 6 | 11 | 25 | 123 | 22 |
| 16 | M | ADHD | 31 | 5 | 5 | 21 | 98 | 19 |
| 16 | M | None | 11 | 0 | 2 | 28 | 95 | 21 |
| 17 | M | OCD | 18 | 10 | 8 | 20 | 116 | 30 |
| 18 | M | OCD | 27 | 5 | 9 | 29 | 116 | 21 |
| 18 | M | OCD | 21 | 19 | 10 | 28 | 113 | 26 |
| 19 | F | None | 30 | 11 | 13 | 26 | 48 | 15 |
| 19 | M | OCD | 27 | 9 | 3 | 22 | 103 | 22 |
| 22 | M | OCD | 22 | 12 | 4 | 22 | 113 | 22 |
| 26 | F | OCD | 24 | 18 | 9 | 29 | 102 | 17 |
| 27 | M | None | 23 | 6 | 12 | 32 | 150 | 34 |
| 40 | M | OCD | 36 | 18 | 15 | 34 | 108 | 18 |
| 41 | F | OCD | 28 | 8 | 11 | 33 | 107 | 34 |

ANOVA of SGI total scores (Fig. 1) revealed a significant effect of diagnosis (F = 16.04; df 1,32; $p <$ 0.0003; TS > HCS) as well as interactions of gender × age group (F = 5.80; df 1,32; $p < 0.02$) and gender × age group × diagnosis (F = 4.48; df 1,32; $p < 0.04$). Importantly, there was a significant interaction of diagnosis × subscale (F = 3.46; df 3,96; $p < 0.02$). Four SGI subscales were previously identified using factor analyses applied to SGI responses from 532 college students[10], reflecting items related to: (1) modulation of stimulus intensity and perceptual inundation, (2) anomalies of focal attention or distractibility, (3) anomalies of radial attention as a result of a low threshold of perception (overinclusion

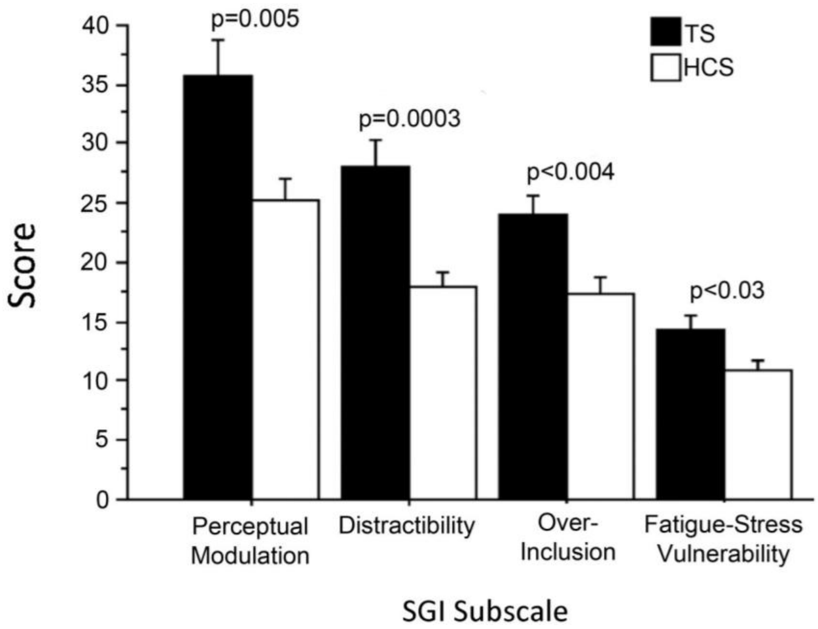

**FIGURE 1.** SGI subscales in TS subjects and HCS. Data are collapsed across age groups and gender. ANOVA detected a significant effect of diagnosis and a significant diagnosis × subscale interaction. Post-hoc significance values for patients vs. controls are shown for each subscale.

and hyperawareness), and (4) vulnerability to perceptual and attentional anomalies during periods of fatigue and stress. Post-hoc comparisons revealed significantly elevated scores in TS subjects on subscales for perceptual modulation ($p = 0.005$), distractibility ($p < 0.0003$), overinclusion ($p < 0.004$), and fatigue-stress vulnerability ($p < 0.03$). "At times I have feelings of being flooded by sounds," and "I have more trouble concentrating than others seem to have" were the two statements most predictive of group separation by Stepwise Discriminant Function Analysis, accounting for 83 and 86% of diagnostic variance in the study sample, respectively. SIAPA was less sensitive to group differences (F = 3.05; df 1,32; ns), and ANOVA did not detect any significant interactions of diagnosis, gender, and age. Despite this, there was a significant positive correlation between SIAPA and SGI total scores ($R^2 = 0.497$; $p < 0.0001$).

No significant relationships were detected between clinical scales and sensory gating measures, nor were scales significantly different between TS subjects with vs. without comorbid OCD. Because SGI subscales were differentially sensitive to diagnosis, separate regression analyses with each subscale were conducted and again failed to detect any significant correlations with clinical scales.

## DISCUSSION

The major aim of this study was to determine whether scales of sensory gating provide novel information regarding TS symptoms. The elevation of SGI scores in TS is a new and robust finding. The SGI was designed to quantify sensory perceptual disturbances in individuals with "psychosis-spectrum" symptoms[10]. Here, TS patients and controls appeared to be most separated by subscales for perceptual modulation, distractibility, and overinclusion. These subscales were developed for use in a different clinical population and, therefore, may not be maximally sensitive for distinguishing TS from comparison groups. Additional analysis identified the most sensitive questions for distinguishing TS vs. HCS groups, related to perceptual modulation and distractibility — features of disorders comorbid with TS such as OCD and ADHD, generally and within this sample. Therefore, the SGI may be detecting a broader sensory gating deficiency and not one specific to TS alone. It is important to note that while the SGI has been validated for use in HCS[10], it has not been validated for use in minors; while the present findings demonstrated no significant simple effect of age on total SGI scores, interaction effects on SGI score were detected between age, gender, and diagnosis. Generally, SGI subscore variability for HCS minors was comparable to that seen in HCS adults (SD range 3.45–10.25 vs. 2.63–8.08, respectively); this was also true in TS minors vs. adults (SD range 6.70–12.46 vs. 3.78–13.21).

Two scales were used to assess clinical symptoms of premonitory or sensory phenomena in TS: the USP-SPS and the PUTS. To our knowledge, no published reports directly compare these scales, and the finding that they are significantly and positively correlated in this sample provides some evidence of internal validity, i.e., that both scales are measuring similar, though not identical, phenomena. That scores in both scales generally tend to increase with age is also consistent with the clinical experience that premonitory events are most often reported among children older than age 10; the age-dependent reporting of premonitory events is thought to reflect the normal development of introspective capabilities, i.e., of bodily awareness, rather than an age-related change in the illness *per se*[5].

The scales used to assess sensory gating and perceptual anomalies appear to detect overlapping sets of information, which diverge as they relate to TS. In other words, while SGI and SIAPA scores were significantly correlated with each other, only SGI scores were elevated in TS. Furthermore, SGI scores were not significantly related to scores in scales of premonitory urges or motor/vocal tics in TS. Previous studies have also reported that SGI and SIAPA do not strongly predict other measures of either sensory or sensorimotor gating[17,18]. These findings suggest that the construct of “sensory gating” as assessed by the SGI is relevant to TS, and that sensory gating deficits in TS reflect processes that are not fully captured by existing scales for motor, vocal, or sensory tics. Presumably, this dissociation at a level of symptomatology reflects separable underlying neural and perhaps genetic substrates, and suggests that the SGI, or some TS-focused derivative of this inventory, might be a valuable phenotype to add to ongoing neuroimaging and genetic studies of TS.

This study is limited by the small sample, containing a heterogeneous group of TS minors and adults, males and females, with a range of comorbid conditions and medications. Nonetheless, the robust findings suggest that the SGI might identify new information about TS that is not detected by existing scales for premonitory symptoms and, therefore, might have value in characterizing the disorder, i.e., via subtyping, assessing treatment response, or identifying different underlying etiologies or patterns of pathophysiology.

## ACKNOWLEDGMENTS/FUNDING/COMPETING INTERESTS

This study was supported by NIMH grant MH59803. There are no competing interests. The authors acknowledge the helpful statistical guidance by Dr. Gregory Light, the valuable input of Dr. Robert McGivern, and assistance in manuscript preparation by Ms. Maria Bongiovanni.
